# Ocular involvement in highly treatment-experienced patients with HIV

**DOI:** 10.22336/rjo.2024.28

**Published:** 2024

**Authors:** Mihaela Cobaschi, Carmen Mihaela Dorobăț, Victor Daniel Dorobăț, Isabela Ioana Loghin, Mioara-Laura Macovei, Adrian Marinescu, Victoria Aramă

**Affiliations:** * “Carol Davila” University of Medicine and Pharmacy, Bucharest, Romania; ** “Prof. Dr. Matei Bals” National Institute for Infectious Diseases, Bucharest, Romania; *** Department of Infectious Diseases, “Sf. Parascheva” Clinical Hospital of Infectious Diseases, Iaşi, Romania; **** Department of Infectious Diseases, “Grigore T. Popa” University of Medicine and Pharmacy, Iaşi, Romania; ***** Department of Ophthalmology, “Carol Davila” University of Medicine and Pharmacy, Bucharest, Romania; ****** Department of Infectious Diseases I, Faculty of Medicine, “Carol Davila” University of Medicine and Pharmacy, Bucharest, Romania

**Keywords:** HIV patients, antiretroviral therapy, cytomegalovirus retinitis, HIV retinopathy

## Abstract

**Introduction:** Ocular involvement in human immunodeficiency virus (HIV) infected and treatment-experienced patients is a significant concern, despite the advancements in antiretroviral therapy (ART) medication. The extended life expectancy of HIV patients has altered the spectrum of HIV-associated ocular diseases, ranging from minor issues to severe vision impairment or blindness. Therefore, understanding these complications becomes crucial in providing comprehensive medical care and quality of life improvement. HIV patients on multiple ARTs can experience various ocular disorders due to the complexity of their treatment regimens, drug toxicities, immune reconstitution, and opportunistic infections. Most worthy to consider are: cytomegalovirus (CMV) retinitis, immune recovery uveitis (IRU), keratoconjunctivitis sicca (dry eye syndrome), and HIV-associated neuroretinal disorders.

**Materials and methods:** A retrospective clinical investigation was conducted on HIV/AIDS-infected patients from January 1, 2013, to December 31, 2023. The study included 62 patients over 18 years, who tested HIV-positive via enzyme-linked immunosorbent assay (ELISA) and confirmed by Western blot (WB), with assessments of HIV plasma viral load (VL) and CD4+ T cell counts (CD4). Data collected included demographics, pathological histories, clinical characteristics, blood tests, assessments for opportunistic infections, patient staging, antiretroviral therapy initiation, and disease prognosis.

**Results:** The study found that of most patients, 37 were aged 30-39 (59.7%), with 59.7% males and 40.3% females. Most had been living with HIV for 10-19 years (35.5%). Initial CD4 counts were < 200 cells/mm3 in 46.8% of patients, which improved to 19.3% when the study was done. CMV retinitis prevalence decreased from 46.8% initially to 35.5% despite ART. Other conditions included ocular toxoplasmosis (3.22%), tuberculosis-related uveitis (1,6%), keratoconjunctivitis sicca (19.3%), and HIV retinopathy (29%). Notably, 62.1% of CMV retinitis patients experienced significant visual acuity reduction. Oral valganciclovir was beneficial for patients with CMV disease affecting multiple sites and effective for both induction and maintenance therapy of CMV retinitis.

**Conclusions:** Managing ocular complications in HIV-experienced patients requires a multidisciplinary approach with regular ophthalmologic evaluations, prompt treatment of infections, and continuous monitoring of ART effectiveness. Early detection and intervention are crucial for preserving vision and improving outcomes. The study highlighted the importance of constant monitoring even after viral suppression.

**Abbreviations:** HIV = Human immunodeficiency virus, ART = antiretroviral therapy, CMV = cytomegalovirus, IRU = immune recovery uveitis, ELISA = enzyme-linked immunosorbent assay, WB = Western Blot, VL = viral load, CD4 = CD4+ T cells.

## Introduction

Ocular involvement in human immunodeficiency virus (HIV) infected and treatment-experienced patients remains a critical area of concern despite advancements in antiretroviral therapy (ART). The evolution of ART has decreased HIV-related mortality over time, but the emergence of drug resistance has created difficulties in managing these patients [**1**]. The prolonged life expectancy of individuals living with HIV has shifted the spectrum of HIV-associated ocular diseases. These patients face various ocular complications that range from minor issues to severe vision impairment or blindness. Understanding these complications is essential for providing comprehensive care and enhancing the quality of life for these patients [**2**].

One of the most severe and vision-threatening conditions in HIV-experienced patients is cytomegalovirus (CMV) retinitis. Despite the widespread use of ART, CMV retinitis remains prevalent, particularly in patients with low CD4+ T cell (CD4) counts. Studies have shown that the prevalence of CMV retinitis in HIV-infected patients can be as high as 20-30% in those with CD4 counts below 50 cells/mm3 though with effective ART, the prevalence has decreased to around 2% in some cohorts. Early diagnosis and prompt treatment are crucial in preventing vision loss associated with CMV retinitis. Additionally, other opportunistic infections such as ocular toxoplasmosis and tuberculosis-related uveitis continue to pose significant risks to these patients, with ocular toxoplasmosis affecting approximately 2% of HIV-infected individuals and tuberculosis-related uveitis being reported in up to 1% of cases [**3**].

The emergence of immune recovery uveitis (IRU) as a complication of ART has added complexity to the management of ocular conditions in HIV-experienced patients. IRU occurs in the immune reconstitution inflammatory syndrome (IRIS) context and is characterized by inflammation that can cause further visual deterioration despite effective viral control. The incidence of IRU has been reported to range from 0.2% to 1.7% among patients receiving ART, with some studies indicating that up to 12% of patients with CMV retinitis may develop IRU. This highlights the need for ongoing ophthalmologic monitoring even after ART initiation [**4**].

Non-infectious conditions such as keratoconjunctivitis sicca (dry eye syndrome) and HIV-associated neuroretinal disorders are also prevalent among HIV-experienced patients. Keratoconjunctivitis sicca, resulting from chronic inflammation and destruction of the lacrimal glands, leads to significant discomfort and potential damage to the ocular surface. It affects up to 20% of individuals with HIV. HIV-associated neuroretinal disorders, including HIV retinopathy and optic neuropathy, result from direct viral effects and immune-mediated damage, affecting approximately 50-70% of patients with advanced HIV disease. Studies have shown that HIV retinopathy alone can be found in 40-60% of HIV-infected individuals, often correlating with the severity of immunosuppression [**5**,**6**].

The prevalence and severity of these ocular conditions correlate with the level of immunosuppression, the duration of HIV infection, and the specific antiretroviral regimens employed. The introduction of highly active antiretroviral therapy (HAART) has significantly reduced the incidence of many opportunistic infections, including CMV retinitis, by as much as 80% in some populations. However, this has also led to new challenges emergence such as IRU. Furthermore, there is a growing need to address the long-term side effects of ART on ocular health, including cataract formation and glaucoma. Studies indicate that HIV-infected individuals are at a 2-4 times higher risk of developing cataracts and glaucoma compared to the general population, with some studies reporting up to a 3.5-fold increased risk for glaucoma [**5**,**7**].

Managing ocular complications in HIV-experienced patients requires a multidisciplinary approach that includes regular ophthalmologic evaluations, prompt treatment of infections, and continuous monitoring of ART effectiveness and side effects. Early detection and intervention are critical to preserving vision and improving overall outcomes. Collaboration between ophthalmologists, infectious disease specialists, and primary care providers is essential to address the unique challenges presented by these patients [**8**].

Recent studies have provided valuable insights into the management and outcomes of ocular conditions in HIV-experienced patients. For instance, oral valganciclovir is an effective treatment for CMV retinitis in HIV patients, with success rates comparable to intravenous ganciclovir, but research on the effectiveness of intravitreal ganciclovir implants for CMV retinitis has shown promising results in preserving vision, with a reported 80-90% success rate in controlling the infection [**9**,**10**]. Additionally, advancements in diagnostic imaging techniques, such as optical coherence tomography (OCT), have improved the detection and monitoring of HIV-associated neuroretinal disorders, allowing for these conditions to have earlier intervention and better management [**10**,**11**].

## Materials and methods

*Database description*

On the foundation of hospital medical data, we conducted a retrospective clinical investigation of patients diagnosed with HIV/Acquired immunodeficiency syndrome (AIDS) in Romania. The study focused on those hospitalized at “Matei Balș” National Institute of Infectious Diseases (INBIMB) and the HIV/AIDS Regional Center at “Sf. Parascheva” Clinical Hospital of Infectious Diseases (RCSPI) in Iaşi. The objective was to highlight the profile and ocular involvement in HIV-experienced patients. The study covered the period from January 1, 2013, to December 31, 2023.

We included patients over the age of 18 years, who were hospitalized at our centers and tested HIV-positive via the enzyme-linked immunosorbent assay (ELISA) test, with confirmation by western blot (WB). Additionally, HIV plasma viral load (VL) and CD4+ T cell counts (CD4) were assessed in these patients. A total of 62 patients met the inclusion criteria for our study.

*Study design*

Data were collected on various aspects of HIV/AIDS patients, including demographics (age and gender), individual pathological histories, clinical characteristics, blood tests (viro-immunological testing), assessments for potential opportunistic infections, patient staging, the initiation and history of antiretroviral therapy, and the course and prognosis of the disease.

The stages of HIV infection were determined using age-specific CD4+ T-lymphocyte counts or the percentage of total CD4 T-lymphocyte cells, based on the guidelines from the Centers for Disease Control and Prevention (CDC) in Atlanta. The progression of HIV infection to AIDS is divided into three stages: Stage 1 is characterized by CD4+ T-lymphocyte levels greater than 500 cells/mm3; Stage 2 has levels between 200 and 499 cells/mm3; and Stage 3 has levels less than 200 cells/mm3. Stages 1 and 2 are classified as HIV infection, while Stage 3 is classified as AIDS.

Serological assessment for those suspected of having HIV involved two ELISA tests, with a Western blot test used to confirm the diagnosis. This work was conducted by epidemiologists from the regional public health management network, after which patients were referred to the local HIV/AIDS facility.

*Study setting*

INBIMB in Bucharest and RCSPI in Iaşi are key medical facilities for infectious disease treatment in Romania. INBIMB is the national reference center for the care of patients with HIV/AIDS infection in Romania, monitoring the largest number of patients with HIV infection. RCSPI serves the Moldova region, has a capacity of 300 beds, and is divided into six pavilions. Pavilion V houses the Infectious Diseases unit and the HIV/AIDS Regional Center, which has 12 beds. Patients at INBIMB and RCSPI are periodically evaluated according to CDC and EACS guidelines.

All blood tests were conducted by the hospital’s central laboratory, with HIV plasmatic viral load and CD4+ T cell counts assessed in the molecular biology lab. HIV viremia was measured using RT-PCR HIV 1 via Cepheid’s GeneXpert®, classifying viral loads as undetectable if below 40 copies/mL and detectable if above 40 copies/mL.

Patients underwent evaluations every six months to ensure adherence to antiretroviral treatment. Each patient had a medical file containing information on coexisting conditions, blood test results, CD4 T cell counts, HIV viral loads, and ART regimens. Data for the investigation were sourced from the paper charts maintained by the center’s patients.

*Statistical analysis*

Data were analyzed using Pearson’s chi-square tests, to examine the relationships between ocular conditions and various HIV-related factors such as CD4 counts and VL. Mann-Whitney tests were employed for non-parametric data analysis.

Demographic Data: Information on age, gender, and HIV infection duration were collected.

## Results

*Patient Characteristics*

The study group included 62 patients with ocular disorders due to ARTs, 38 of whom were selected from INBIMB and 24 from RCSPI.

**Fig. 1:**
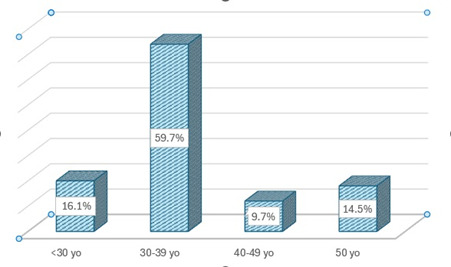
Distribution of patients with ophthalmological disorders according to age group

Based on their age groups, most of them were between 30-39 years old (59.7%), followed by < 30 years old (16.1%), 40-49 years old (9.7%), and ≥ 50 years old (14.5%) (**Fig. 1**). 

**Fig. 2:**
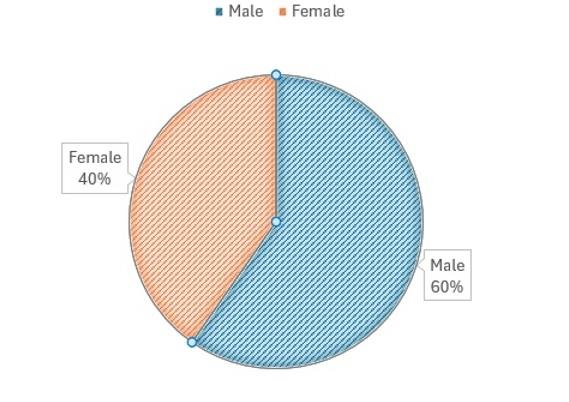
Distribution of patients with ophthalmological disorders according to gender

Based on their gender: 59.7% were males and 40.3% were females, thus showing a predominance of the first (**Fig. 2**).

**Fig. 3:**
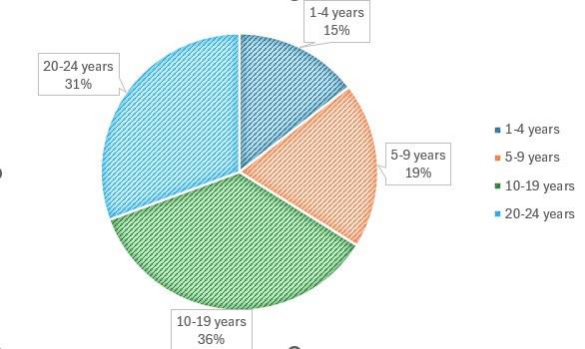
Distribution of patients with ophthalmological disorders and HIV+ status

Based on the length of time since HIV+ status: most patients were HIV+ for 10-19 years (35.5%), followed by 20-24 years (30.6%), 1-4 years (14.5%), and 5-9 years (19.4%). All of them had ocular impairments (**Fig. 3**).

**Fig. 4:**
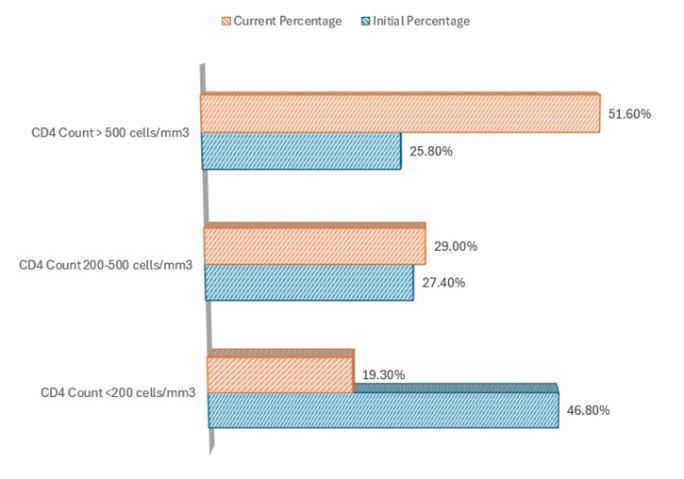
Distribution of patients with ophthalmological impairment according to CD4 count

**Fig. 5:**
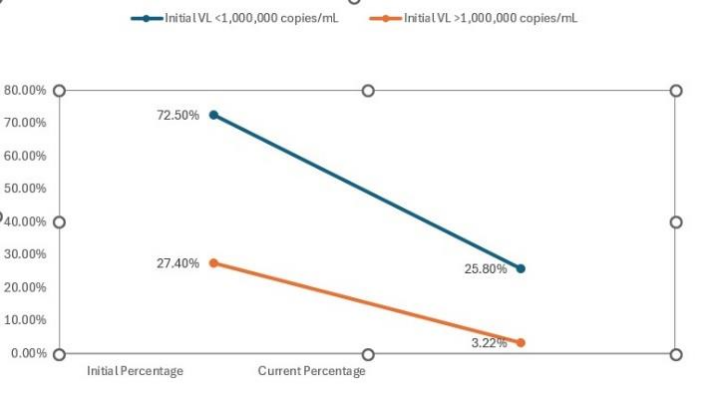
Distribution of patients with ophthalmological manifestations according to VL

Considering CD4 counts and VL, initially, 46.8% had CD4 counts < 200 cells/mm3, 27.4% had 200-500 cells/mm3, and 25.8% had > 500 cells/mm3. CD4 counts when the study was undergone showed improvement with 19.3% < 200 cells/mm3, 29.0% between 200-500 cells/mm3, and 51.6% > 500 cells/mm3 (**Fig. 4**). Initial VL was < 1,000,000 copies/mL in 72.5% and > 1,000,000 copies/mL in 27.4% (**Fig. 5**).

*Ocular disorders*

**Fig. 6:**
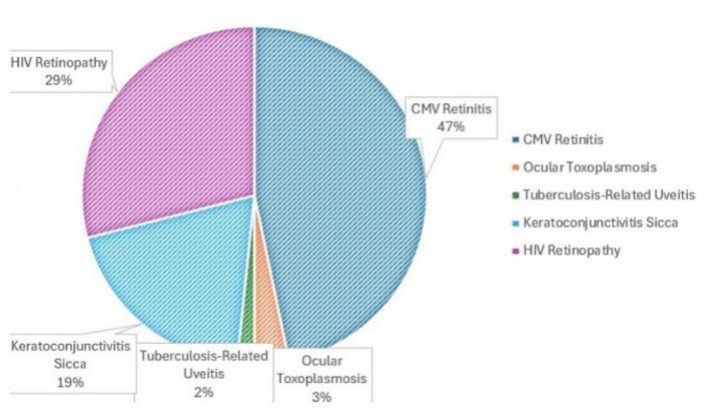
Distribution of patients with ophthalmological changes according to the type of ocular impairment

The prevalence of CMV retinitis decreased from 35,5% initially to 29,0% when the study was done. This condition is particularly found among patients with CD4 counts below 50 cells/mm3, though ART has reduced its overall incidence to around 2% in some cohorts. Among the 62 patients, 29 (46.8%) initially presented with CMV retinitis, and despite ART, 22 (35.5%) still showed signs of the condition. Ocular toxoplasmosis affected approximately 3.22% of the cohort, with 1-2 patients diagnosed during the study period and tuberculosis-related uveitis were present in up to 1.6% of patients, equivalent to about 1 patient in this cohort. Keratoconjunctivitis sicca affected up to 19.3% of individuals with HIV infection. HIV retinopathy alone was present in 53.2% of HIV+ individuals, often correlating with the severity of immunosuppression, thus becoming the commonest ocular lesion (**Fig. 6**). Out of a total of 62 patients with multiple ophthalmological impairments, 3 patients (4.83%) who responded favorably to local treatment presented IRU.

*Specific findings*

Considering the visual acuity, most patients with CMV retinitis reported a significant decrease in visual acuity, with 18 out of the 29 affected patients experiencing a reduction to 20/200 (severe visual impairment) or worse (62.1%). After changing ARTs and initiating oral Valganciclovir 900 mg BD for 21 days followed by 900 mg daily for at least 6 months, only 5 patients (22.7%) clinically had visual improvements and gained 2-3 lines in best corrected visual acuity, 5 patients (22.7%) experienced altering of vision, while 12 patients (54.5%) had no changes. Regarding the patients who only had HIV retinopathy, vision was maintained, and retinal lesions were not present at 6-12 weeks of ART switch, with an average of 7.2 weeks.

## Discussion

For all 62 patients, ART therapy regimens were established according to the range of available drugs at the time of diagnosis, existing guidelines, and considering possible adverse events and drug interactions. Of the 62 patients, 31 were part of the pediatric cohort, who started treatment with ART regimens specific to the 90s, efavirenz + nucleoside reverse transcriptase inhibitor (NRTI)/ non-nucleoside reverse transcriptase inhibitor (NNRTI) or azidothymidine monotherapy or combination therapy, with the regimens later being updated, reaching 7, 8, or 9 treatment regimens. The rest of the patients mostly started with a protease inhibitor (PI) and NRTI or NNRTI (atazanavir /ritonavir, tipranavir /ritonavir, lopinavir /ritonavir, darunavir /ritonavir) or integrase inhibitor (INI), with some of them later switching to a modern “one single tablet” (OST) regimen (34 patients, 51.6%). Among the patients we evaluated, some followed the same therapeutic regimen (efavirenz +NRTI) for a long time, even 10 years. Following the evolution of patients in the study, we can consider that even in cases of regimens with highly potent antiviral action, PI + NRTI/NNRTI, or PI + INI + NNRTI (etravirine), the occurrence of ocular disorders was due to the long-term progression of the disease or associated opportunistic infections. It should be noted that although there were adherent patients, those with many treatment regimens (over 5) might have experienced “therapeutic fatigue” over time, even though all patients in the study received psychological support. Nevertheless, therapeutical pauses were taken, which undoubtedly favored the occurrence of complications, including ophthalmological ones. When the study was made, the evaluations included the mathematical statistical assessments, correct ART therapy administered early, and an adherent patient’s prolongation of average life expectancy implicitly delaying the onset of complications. Over time, due to the pathophysiological mechanism characteristic of HIV’s action in the body and its remarkable ability to develop resistance, immunosuppression became dominant, necessitating multidisciplinary assistance.

The perspective was undoubtedly directed towards an easily administrable regimen, with high potency and resistance threshold to enhance the patients’ quality of life, thus reducing late-onset complications.

The data indicated a significant prevalence of ocular complications among HIV-experienced patients. CMV retinitis remains a critical concern, especially in those with low CD4 counts. Despite ART, opportunistic infections and non-infectious conditions continue to affect ocular health.

Lower CD4 counts and higher VLs correlate with increased ocular complications. Patients with CD4 counts < 200 cells/mm3 were more likely to develop severe ocular issues, emphasizing the need for regular monitoring and early intervention. Statistical analysis showed a significant association (p < 0.05) between low CD4 counts and the prevalence of CMV retinitis, highlighting the vulnerability of patients with severe immunosuppression. The improvement in CD4 counts over the study period demonstrated the effectiveness of ART in immune recovery, although challenges like IRU and other ocular complications persisted.

Immune Recovery Uveitis (IRU), a complication of ART, has added complexity to ocular management. IRU incidence ranged from 0.2% to 1.7% among ART recipients, with some studies indicating up to 12% in those with CMV retinitis. Among the study cohort, 5 patients (8.1%) developed IRU after initiating ART, underscoring the need for vigilant ophthalmologic monitoring even after viral suppression was achieved.

## Conclusion

Managing ocular complications in HIV-experienced patients requires both infectious disease and ophthalmology specialists. Regular ophthalmologic evaluations, prompt treatment of occurring co-infections, and continuous monitoring of ART effectiveness and side effects are crucial. Thus, collaboration becomes essential in addressing the unique challenges presented.

Early detection and intervention are critical to preserving vision and improving overall outcomes. The study highlighted the importance of continuous monitoring, even after achieving viral suppression, due to the risk of immune recovery uveitis (IRU) and other complications.

Future research should focus on long-term outcomes and the development of targeted therapies to address specific ocular complications in HIV infected experienced patients. Additionally, education and awareness programs for both healthcare providers and patients can help in early recognition and management of these conditions, ultimately improving the quality of life for HIV+ individuals.

**Conflict of Interest Statement**

The authors state no conflict of interest. 

**Informed Consent and Human and Animal Rights Statement**

Informed consent has been obtained from the individuals included in this study.

**Authorization for the use of human subjects**

Ethical approval: The research related to human use complies with all the relevant national regulations and institutional policies, as per the tenets of the Helsinki Declaration, and has been approved by the review board of “Prof. Dr. Matei Balş” National Institute for Infectious Diseases, Bucharest (No. 2211/ 12.02.2019). 

**Acknowledgments**

None. 

**Sources of Funding**

None. 

**Disclosures**

None.
